# Effects of Exercise on Urinary Parameters and Proteins in Well-Trained Canicross Dogs: A Preliminary Study

**DOI:** 10.3390/ani14223216

**Published:** 2024-11-09

**Authors:** Giuseppe Spinella, Simona Valentini, Thomas Dalmonte, Gloria Isani, Giulia Andreani

**Affiliations:** Department of Veterinary Medical Sciences, Alma Mater Studiorum—University of Bologna, Via Tolara di Sopra 50, Ozzano dell’Emilia, 40064 Bologna, Italy; giuseppe.spinella@unibo.it (G.S.); thomas.dalmonte2@unibo.it (T.D.); gloria.isani@unibo.it (G.I.); giulia.andreani2@unibo.it (G.A.)

**Keywords:** canicross, injury prevention, clinical examination, urinary biochemical parameters, urinary proteome, albuminuria

## Abstract

The aim of the present study was to investigate the effects of exercise during canicross competition on urinary biochemical parameters and urinary proteins. Our results showed that, among the parameters analysed in routine urinalysis, pH was affected by exercise and albuminuria also showed changes immediately after physical exercise with a return to baseline within 2 h after the physical activity. In particular, SDS-PAGE electrophoresis allowed the separation of the urinary proteins on the basis of their molecular mass allowing a qualitative evaluation of the urinary proteome and a quantitative analysis of urine albumin and uromodulin, with a significant increase in urinary albumin and a decrease in uromodulin after exercise. In this study, urine was collected by spontaneous voiding and the use of this non-invasive sampling method proved useful in obtaining information on the health of the animals in the immediate moments before and after the competition without increasing the stress of the animals involved in the competition by any visits or invasive manipulations by veterinarians.

## Simple Summary

The aim of this prospective study was to determine whether competition-associated exercise and stress could have a negative effect on urinary biochemical parameters and urinary uromodulin and albumin separated using SDS-PAGE electrophoresis in adequately trained dogs. The results showed that urine pH was affected by exercise and proteinuria levels changed immediately after physical exercise with a return to baseline within 2 h after the physical activity; moreover, quali-quantitative evaluation of the urinary proteome showed a significant increase in urinary albumin and a decrease in uromodulin after exercise. Urinalysis and the evaluation of the urinary proteome by SDS-PAGE electrophoresis proved to be a non-invasive and economic technique for investigating the health status and pre- and post-exercise changes in athletic and working dogs.

## 1. Introduction

Unlike high impact sports like flyball and agility, canicross is an endurance dog sport that is becoming increasingly popular in Italy and in other European countries [1]. The other sport that falls into the endurance category is dog sledding, which has a different impact because it takes place over longer distances and in more severe environmental conditions. In canicross, a dog and a human run together: the dog is attached to the runner’s waist with canicross harness, bungee line or canicross belt. Depending on the equipment used, canicross can be subdivided into other disciplines such as bike joring and scooter joring. Any breeds of dogs may be involved in this activity, but the most commonly used are German Shorthaired Pointer, English Pointer, Belgian Malinois, Border Collie, Beauceron, and mixes such as hounds (crossbred from the Alaskan Husky and a similar-looking pointer), Alaskan Huskies and Greysters (crossbred from the Greyhound and a similar-looking pointer). The distance of canicross races ranges from relatively short “sprint” distances of about 5 km to longer competitive distances of 45 km or more [1,2].

A relatively small number of papers have investigated the correct training or the incidence of injuries in dogs competing in this discipline [1,2]. In 2018, Lafuente and Whyle [1], reported that canicross is a quite safe sport for dogs compared to the other most popular canine disciplines (agility and flyball); indeed, the authors reported that only 21.9% of dogs had experienced at least one injury [1]. The incidence was generally lower than other high impact disciplines: 32–41.7% for agility [3,4,5] and 34–39% for flyball [6,7,8]. The most common injuries reported were: lacerations, abrasions and punctures, most frequently occurring in the footpads of the forelimb [1]. More recently, in 2022, Erjavec et al. [2] have investigated the health status of seven trained canicross dogs that were subjected to two acute exercise episodes during the training. For this specific investigation the following parameters were assessed: rectal temperature, hematological and biochemical parameters, as well as blood oxidative stress parameters before and during a two-day canicross training session and after a 24-h rest period [2]. The hematological parameters showed limited changes, remaining within the reference ranges. No significant differences in oxidative stress parameters were found between any of the sampling times. The authors concluded that the trained canicross dogs included in their study were in a good physical condition and correctly trained [2]. Indeed, it is commonly assumed that well-trained athlete can be defined as the athlete who performs his/her activity consistently and effectively with the least effort.

The blood parameters useful for assessing the systemic effects of physical exercise are not easily monitored during competition; therefore, it could be very useful to identify a non-invasive biological matrix. Urine is an interesting alternative sample for investigating the health status and pre- and post-exercise changes in athletic and working dogs. Urine has recently been studied in military working dogs and albuminuria was found to be an early biomarker for monitoring renal function during training sessions [9].

The aim of this prospective study was to investigate the effects of exercise on urinary biochemical parameters and urinary proteins separated using SDS-PAGE electrophoresis in well-trained canicross dogs before and after an Italian national 5-km competition.

## 2. Materials and Methods

The study was carried out according to European Union Directive 2010/63/EU and was approved by the Animal Welfare Committee of the University of Bologna (Project ID 914). The investigation was conducted during the national competition of 5-km canicross race in Trecenta (RO), Italy. All dog-owners participated to the study on a voluntary basis and signed an informed consent form regarding research participation. All dogs included in the study had regularly participated in national competitions and carried out continuous training. Twelve female dogs were included in this study: five Eurohound dogs, three Weimaraner, one Greyster, one Border Collie, one Siberian Husky and one Alaskan Malamute. The mean age was 5.6 ± 2.1 years: this value has been fixed as cutoff in order to divide dogs in “young adult” (≤5 years, 7 dogs) and “old adult” (>5 years, 5 dogs). The mean age of young adult was of 3.1 ± 1.0 years, while the mean age of old adult was of 6.7 ± 1.5 years. The mean weight was 25.5 ± 4.4 kg for the whole group (25.8 ± 3.1 kg for young adult and 25.0 ± 5.9 for old adult dogs).

Seven females were entire (4 young adult and 3 old adult) and five were spayed (2 young adult and 3 old adult).

The dogs followed different diets set by their handlers, but they were fasted at least 2 h before competition, with the exception of energy supplements administered 1–2 h before the competition for only one dog, previously checked to exclude any influence on results.

Before the competition race, all dogs were submitted to a signalment and physical examination (including pulse rate evaluation) by licensed veterinary doctors to ensure the current healthy status. No dogs received any medication with steroids or non-steroidal anti-inflammatory drugs.

Ten ml of midstream urine was collected by spontaneous voiding into sterile urine cups by a veterinary doctor wearing gloves at three different times: T0 (at rest, before competition, fasting for at least 2 h), T1 (first urinary voiding after competition) and T2 (two hours after activity). Three of the 12 dogs in the study did not return for the T2 check-up.

### 2.1. Urinalysis

All the urine samples were kept refrigerated (+4 °C) and were processed on a routine basis within 2 h after collection. In particular, the urinalysis consisted of a macroscopic examination evaluating the colour and turbidity. Urine specific gravity (USG) was measured using a manual refractometer (Bormac, Modena, Italy), and the chemical evaluation was carried out using a semi-quantitative dipstick test (Combur10Test, Roche Diagnostic, Mannheim, Germany). After centrifugation at 1500× *g* for 10 min, urine sediment was observed under both high (400×) and low microscopic fields (100×). Urine supernatants were divided into aliquots and stored in part at −20 °C for a maximum of 7 days for total proteins and creatinine determination, and in part at−80 °C for the subsequent proteomics analysis.

### 2.2. Urine Protein to Creatinine Ratio

Urine total proteins (uTP) and creatinine (uCr) were measured as reported by Spinella et al. (2023) [9] using commercial kits (Urinary/CSF Protein, OSR6170, and Creatinine OSR6178, Olympus/Beckman Coulter, Atlanta, GE, USA) on an automated chemistry analyzser (AU 480, Olympus/Beckman Coulter, Atlanta, GE, USA). The urine protein:creatinine ratio (UPC) was calculated using the following formula: UPC = uTP (mg/dL)/uCr (mg/dL).

### 2.3. One-D-Electrophoresis

After thawing and centrifugation at 3000× *g* for 10 min, the urinary proteins in the supernatants were separated using a sodium dodecyl sulfate-polyacrylamide gel electrophoresis (SDS-PAGE) 4–12% system (NuPAGE, Thermo Fisher Scientific, Waltham, MA, USA) as previously described [10]. SDS-PAGE is commonly used to obtain high-resolution separation of complex mixtures of proteins. The method initially denatures the proteins which will undergo electrophoresis and then separates them based on their molecular mass. Briefly, three µg of protein were loaded on 4–12% polyacrylamide gel in MOPS buffer with SDS (Thermo Fisher Scientific, Waltham, MA, USA). If the uTP concentration was lower than 0.100 µg/µL, urine was concentrated using spin columns with a molecular mass cut-off of 3 kDa (Vivaspin 500, Sartorius, Goettingen, Germany), following the manufacturer’s instructions. Each gel was also loaded with standard proteins of known molecular mass (Precision Plus Protein Standard, Biorad, Hercules, CA, USA). The gels were stained with Coomassie brilliant blue (PageBlu protein staining solution; Thermo Fisher Scientific, Waltham, MA, USA). After staining, each gel was digitalized (ChemidocMP, BioRad, Hercules, CA, USA), and pherograms were obtained using commercial software (ImageLab 5.2.1, BioRad, Hercules, CA, USA). The concentration of uromodulin and albumin in the respective bands was calculated using an internal standard of quantity as reported in [11] and their ratios with creatinine (uUC and uAC), were calculated using the following formulas:

uUC (without unit) = urinary uromodulin (mg/dL)/uCr (mg/dL)

uAC (without unit) = urinary albumin (mg/dL)/uCr (mg/dL)

To better separate the low molecular mass (LMM) bands, two μg of protein for each sample were also loaded on 12% polyacrylamide gel in MOPS buffer with SDS (Thermo Fisher Scientific, Waltham, MA, USA) and electrophoresis was carried out following the procedure reported previously; gels were stained with silver nitrate (SilverQuest; Thermo Fisher Scientific). After staining, the gels were digitalized (ChemiDoc XRS+ system; Bio-Rad) and the electropherograms were obtained (ImageLab 5.2.1 software; Bio-Rad). As an internal standard, 10 ng of protein obtained from a solution containing 10 ng/µL of lactate dehydrogenase (LDH) (Sigma-Aldrich, Darmstadt, Germany) were added to each sample. Qualitative evaluation of protein profiles was performed by the calculation of the total number of bands in each sample, presenting a volume of the band greater than half the volume of the internal standard.

### 2.4. Statistical Analysis

The distribution and homoscedasticity of the variables in each group over time were assessed using the Shapiro-Wilk and Levene tests, respectively. If the distribution was not normal, the Friedman test was applied followed by the Nemenyi post-hoc test. Conversely, if the distribution was normal and the data had equal variances, ANOVA for repeated measures was performed. In addition, the Mauchly’s test for sphericity was applied and, if sphericity was not respected, the *p*-value of ANOVA was corrected using the Greenhouse-Geisser method.

Furthermore, to assess the effect of age on the variables, subjects were split in young adult (A) and old adult (S) dogs (the cut off was set at 5 years of age). T-test and Mann-Whitney test were performed for normal and not-normal distribution, respectively.

For variables related to proteinuria, the missing values at T2 were predicted by linear multiple regression for uromodulin, uTP, uCr and a linear simple regression model for albumin, respectively [12]. The distribution and homoscedasticity of the residuals were assessed using the Shapiro-Wilk and Breusch-Pagan tests, respectively. In addition, the residuals of each model were evaluated through the visualization of ordinary least squares (OLS) plots visualisation. Multicollinearity was assessed by calculating variance inflation factor (VIF) coefficients. and values <5 were considered suitable for model formulation [13]. The F-test of overall significance in regression was used to assess whether the linear regression model provided a better fit to the dataset than a model without predictor variables [12]. Finally, the goodness-of-fit was assessed using R^2^ adjusted and R^2^ for linear multiple regression and linear simple regression models, respectively [12]. The analysis and assessment of model characteristics are reported in Table S1 as Supplementary Materials.

In order to point out the proportion of albuminuria cases in dogs after training (T1) and after the recovery period (T2), the cumulative incidence was calculated with the following Equation (1) [14].

Cumulative incidence = new cases of microalbuminuria (T0-T1 or T0-T2)/population at risk (all dogs).(1)

A cut-off of uAC (*1000) ≥30 was chosen to detect albuminuria as reported by [15].

Spearman correlation was used to assess the correlation between variables related to proteinuria (uromodulin, albumin, uTP, uCr, uUC, uAC, UPC) and age expressed in years at each time point [14].

Statistical analyses were performed using R 4.3.2 (R foundation for statistical computing; Vienna, Austria; https://www.R-project.org/, accessed on 21 March 2024). A *p*-value < 0.05 was considered statistically significant.

## 3. Results

Twelve healthy canicross dogs (7 entire and 5 spayed females) of different breeds and age were included in this study. The pre-competition mean pulse rate was of 79 ± 24.4 beats per minute.

### 3.1. Urinalysis

The colour of the urine samples ranged from pale yellow to yellow and did not change during the study. The turbidity ranged from clear to slightly cloudy at T0, but was clear in all samples at T1 and T2. Complete urinary data are reported in Table 1. At T0, urine pH had a mean value of 6.6 ± 0.3 and was significantly lower (*p* < 0.01) compared to pH at T1 and T2 (8.1 ± 0.2 at both times). In contrast, the USG at T0 was 1051 ± 6, higher than USG measured in urine collected at T1 (1041 ± 6) and T2 (1042 ± 7). The semiquantitative dipstick test was positive for proteins (30–100 mg/dL) in nine samples at T0 and in ten at T1 and T2 with no significant differences among sampling times; only one sample was positive (17 mg/dL) at T0 and T2 for bilirubin and at T0 and T1 for urobilin. The dipstick test was positive for glucose in five samples at T0 and T1 and in seven urines at T2, but negative for ketones in all samples. Finally, three samples were positive for erythrocytes (two at T0 and one at T1) and for leukocytes (two at T0 and one at T2).

Microscopic evaluation of the urine sediment showed occasional casts (hyaline and granular), RBCs, WBCs and soil contaminants such as pollen, mould spores, bacteria and vegetal fibres. In contrast, crystals were found in five urines sampled at T0 (struvite in three samples and calcium oxalate in two samples) and two at T1 and T2 (struvite). No epithelial cells were found in the urine sediments.

Data on uCr, uTP and UPC are reported in Table 2. The mean values at T0 of uCr and uTP were 255 ± 43 and 20.3 ± 3.3 mg/dl respectively and the variations among sampling times were not significant; otherwise UPC at T0 showed a value of 0.080 ± 0.005 significantly (*p* = 0.04) lower than that measured in urines sampled at T1 of 0.128 ± 0.14.

### 3.2. Urine Protein Electrophoresis

**Figure 1:**
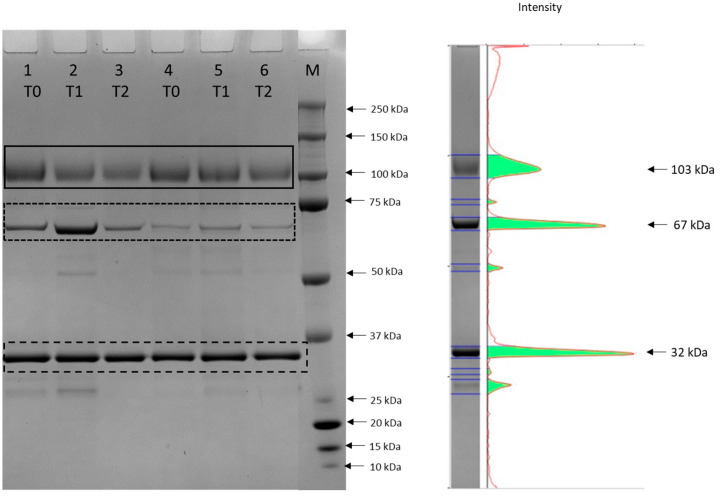
Representative SDS-PAGE 4–12% gel of the proteins in urine sampled respectively at T0, T1 and T2 from an old adult (lanes 1, 2, 3) and a young adult dog (lanes 4, 5 and 6). The black solid box indicates uromodulin (103 kDa), the black dotted box indicates albumin (67 kDa), and the black dashed box indicates the internal standard of quantity (1 μg). A molecular mass marker (M) was also loaded. A representative pherogram (lane 2) is reported on the right.

**Figure 2:**
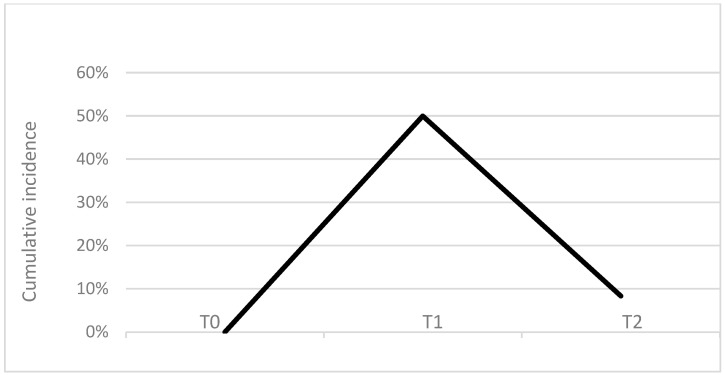
Cumulative incidence of microalbuminuria cases reported in percentage at the different sampling times.

A representative 4–12% gel and a pherogram of urine samples are reported in Figure 1. The urine samples analysed in the present research showed a common pattern of protein bands similar to those reported in healthy dogs by [11], and characterized by the presence of two evident bands with apparent molecular masses of 103 and 67 kDa superimposed on those identified by [11] as uromodulin and albumin using mass spectrometry. The intensity of the albumin band was more pronounced in the post-exercise urines collected at T1. The urine sampled before exercise showed the highest concentration of uromodulin (9.3 ± 1.5 mg/dL), statistically significant (*p* = 0.012) with respect to the urine sampled at T2 (5.0 ± 0.9), while for uUC there was a decrease between the value measured in the urine sampled at T0 and those sampled at T1 and T2, but without significant differences. For albumin, the highest concentration was found in urine collected at T1 (7.7 ± 2.6 mg/dL) with a significant difference compared to T0 (1.5 ± 0.5 mg/dL) (*p* = 0.0044) and T2 (2.0 ± 0.7 mg/dl) (*p* = 0.0086); the same differences were also present for uAC (*p* = 0.00008 and *p* = 0.029 respectively). The cumulative incidence of microalbuminuria cases is reported in Figure 2 and represents 50% of the total cases in dogs after training at T1 and to 8% in dogs after the recovery period at T2.

**Figure 3:**
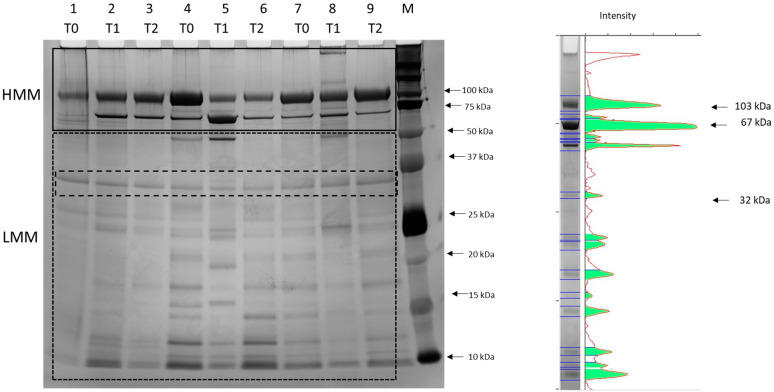
Representative 12%SDS-PAGE gel stained with silver staining of the proteins in urines sampled respectively at T0, T1 and T2 from two adult dogs (lanes 1, 2, 3 and 7, 8 and 9) and a young adult dog (lanes 4, 5 and 6). The black solid box indicates HMM (high molecular mass) proteins, the black dotted box LMM (low molecular mass) proteins and the black dashed box an internal standard. A molecular mass marker (M) was also loaded. A representative pherogram (lane 5) is reported on the right.

To investigate whether exercise induces qualitative changes in the urinary low-abundance and low molecular mass (LMM) proteins, all the samples were also loaded on 12% SDS PAGE gels and stained with silver staining which has an excellent sensitivity in the nanogram range; a representative gel is reported in Figure 3. The pattern of bands with molecular masses >50 kDa (high molecular mass HMM) was similar in all the samples with two more evident bands at 103 and 67 kDa containing uromodulin and albumin, as observed in gels stained with Coomassie, and an additional band at 74 kDa, probably related to transferrin as reported in cats by [10]. The bands with molecular masses <50 kDa (LMM) were present in all the samples and most of them had a common profile with bands at 24, 21, 17, 14, 12 and 10 kDa. The number of LMM protein bands is reported in Table 3 and was 9.1 ± 0.8 at T0 with a significant increase after exercise at T1 (*p* = 0.02) and a significant decrease at T2 compared to T1 protein (*p* = 0.01).

When considering the differences between spayed and intact females no significant differences were found between the groups and no differences were found even between young adult and old adult dogs at the same time either for urinary analytes (Table S2) or for concentrations of total proteins, creatinine, albumin, uromodulin and their ratios (Table S3).

Finally, Table S4 shows the *p*-values obtained with the Spearman correlation analysis between the variables related to proteinuria (uromodulin, albumin, uTP, uCr, uUC, uAC, UPC) and age expressed in years at each time point. Two significant positive correlations were present at each time point, specifically between albumin and uAC and between uTP and uCr. Before exercise a significant negative correlation was found between age and uUC, while a positive correlation was found between age and UPC. After exercise at T1 many positive significant correlations were found: uromodulin correlated with uTP and uCr, uUC with UPC and finally UPC with albumin and uAC. Similarly, after exercise at T2 there were positive significant correlations between uromodulin and uUC, uTP and UPC and one negative correlation between age and uUC.

## 4. Discussion

In this study, all included dogs were routinely trained for canicross activity and all recorded physiological parameters excluded any macroscopic alterations, that might allow the exclusion from competition. The mean pre-competition pulse rate was 79 ± 24.4 beats per minute. This parameter is commonly evaluated during the clinical examination and identification of dogs before the competition. Previous papers have reported similar values in working dogs [16], while pulse rates were slightly lower in agility and sled dogs [17,18], probably related to different environmental conditions and types of exercise that results more similar to the condition observed in working dogs. The clinical evaluation was carried out in a quiet environment away from the start or competition area to provide a more objective evaluation, as it has been observed that pulse rate in working and sports dogs could increase in the pre-activity phase due to the anticipatory response to the competition-related excitement [16,19,20].

Our decision to include only female dogs in the study was suggested by previously published results in order to obtain a more homogeneous group and to avoid possible physiological differences in the urinary proteome due to sex [9,21]. In particular, the physiological presence of two additional bands at the apparent MM of 18 and 12 kDa, identified as arginine esterase in the urine from entire male dogs [9] causes an increase in proteinuria measured as UPC and consequently also in the USG compared to neutered male and female dogs. On the other hand, the entire/spayed status of the 12 females included in this study did not show a significant effect on the variables considered, as summarized in Tables S3 and S4. Similarly, the age variable did not determine a significant difference in the parameters analyzed between the two groups of young adults and old adults at any of the times considered. Furthermore, no significant difference was found between the three time points in the subgroups for any of the parameter analyzed, probably due to the small sample size of subjects included in the subgroups themselves.

Among the parameters evaluated in urinalysis, the USG and the urine pH directly reflects the hydration status and the ability of the renal tubules to concentrate urine; under physiological conditions, intra- and inter-individual variations of these parameters are influenced by various biological factors such as age, sex, drinking avidity, micturition frequency, dietary moisture content, fasting status and the activity level and by environmental factors such as the temperature and humidity. Variations in USG and pH could represent a response to the stress for renal and muscular function and their monitoring can be considered an important and immediate approach to evaluate health status in athlete dogs. In this scenario, the interpretation of USG for an individual dog could be difficult, as the same values might be indicative of disease or simply due to individual variations; Current guidelines recommend performing USG assessment on urine collected in the morning when the animal is fasting, as this is likely to represent the most concentrated USG for that animal throughout the day [22]. However, other authors did not find a significant difference in USG between the first morning or afternoon urine, although temporal variations are reported [23]. The USG value determined in this study at T0 was similar to those reported in the literature for healthy dogs [22,23]. The same authors report a decrease in urinary USG in dogs with each increasing year of age [22,23]; however, in our study no significant difference in USG at T0 was observed when considering the two subgroups of young and old adults, although USG was slightly lower in the group of older dogs. After the competition, the USG decreased at both T1 and T2 compared to T0. This decrease could be related to the fact that the animals had different watering points after the competition, whereas the urine at T0 was collected in the early morning when the animals were resting and fasting. Furthermore, in their study, Van Vonderen et al. (1997) [24] observed a significant difference in USG between the first morning urine and the afternoon urine, suggesting consistent temporal differences in USG, regardless of the physical exercise performed and the availability of water to drink.

The urine pH at T0 in all subjects was within the range (5.0–7.5) reported by several authors [9,25] for healthy dogs, with little variation between dogs. Despite the dynamic nature of urine pH, the uniformity in urine pH at the three time points was expected, because the dogs enrolled in the study were all healthy based on physical examination and urinalysis, and their owners reported no signs of urinary tract disease in the 6 months before and after the competition. In addition, urine samples were always collected early in the morning, by spontaneous voiding and from fasting animals; samples were also analyzed within 2 h of urination and kept on ice until analysis to limit any pH changes due to sample storage.

The urinary pH measured at T0 was significantly lower compared to T1 and T2, reaching a maximum value of 8.1 ± 0.2 after the competition. The values at each time point were also similar in all subgroups considered, regardless of age and sterilization. The increase in urine pH after physical exercise has also been reported by other authors after a 5 km run at 25 °C [26] or after a search training session in military dogs [9]. On the other hand, a decrease in pH to ≤5 after physical activity could indicate lactic acidosis and hypohydration [27] in dogs that have not been adequately trained for intense exercise. The other urinary analytes determined using a semiquantitative dipstick test did not show any significant differences.

For the biomarkers of proteinuria, the absence of three data at T2 posed a problem because the statistical tests used in this study, such as Friedman’s test and ANOVA for repeated measures, use a listwise deletion, which requires an equal sample size at each time point as an assumption. For these reasons, and considering that the aim of the study was to evaluate possible changes in the urinary proteome after exercise, the authors decided to use simple regression and multiple regression to replace missing values and obtain a complete dataset at T2. As reported by Daniel et al. [12], one of the main advantages of regression is in the field of prediction, which allows obtaining a prediction equation for a most likely value of Y for the determined values of X [12]. Considering the strict relationship between the X values and the predicted Y, a complete dataset allows the application of a statistical analysis supported by a larger sample size and, thus, providing results paired with a high statistical accuracy and power.

Regarding proteinuria, numerous differences were found between sampling times for UPC, urinary albumin and uAC and the number of bands, with values at T1 significantly higher than those at T0 and T2; while for urinary uromodulin, values at T0 were significantly higher than those at T2. The assessment of proteinuria by UPC, which correlates with 24-h protein loss, has been reported by authors extensively [9,28,29]. Bitches included in the study were non proteinuric before and after the competition according to the 0.2 cut-off reported by IRIS (International Renal Interest Society) [30] and also had significantly higher UPCs after the exercise at T1, indicating an increase in proteinuria that was not detected using the dipstick test. Moreover, a positive correlation was found between age and UPC at T0 (Table S4), which may be due to the fact that older dogs tend to present changes in renal function, as reported by other authors [29]. The proteinuria post-exercise seems to be influenced by the intensity of the effort [27]. If moderate exercise could cause an increase in the glomerular filtration, during intense efforts a mixed proteinuria could arise due to excessive glomerular filtration of HMM proteins and a low tubular reabsorption of LMM proteins [26,28].

In this study, SDS-PAGE electrophoresis was used for the qualitative and quantitative analysis of proteinuria. This electrophoretic method has some important advantages, including the fact that no antibodies are used and the possibility of analysing the concentration of different proteins in the same analytical session, resulting in lower analytical costs. In addition, the method is easy to perform and does not require specialized equipment, so it could be included in the clinical analytical routine. Urine albumin and uAC showed a similar trend over time. The calculation of uAC, based on urine creatinine levels, could be influenced by muscular mass and strength, in addition to renal function. Furthermore, in our study albumin and uAC were always significantly correlated and showed the same significant differences between the sampling times. For this reason, we believe that the dogs included in the study could be considered adequately trained for the competition as there are no variations in urinary creatinine concentration due to the effort, without affecting the calculation of albuminuria as uAC. Albuminuria is rarely used as a diagnostic marker to determine renal function in dogs and is usually measured by an immunoturbidimetric method based on monoclonal antibodies against human albumin [28,29,31] and is therefore not species specific for species of veterinary interest.

Falus et al. (2022) [31] reported a reference interval for UAC of 0.019 in clinically healthy dogs, with no differences due to age, breed, sex or body weight. In healthy humans the upper physiological reference limit for uAC is slightly higher at 0.03, and this value has been accepted in dogs by several authors [15,28]. This threshold was used in our study to calculate the cumulative incidence (Figure 2) of subjects exceeding the threshold at T1 (50%) and at T2 (8%). This result clearly indicates that moderate endurance activity such as the described canicross competition, can induce transient proteinuria, that resolves spontaneously after one hour. The mechanisms reported by other authors to explain transient proteinuria after exercise are diverse and include hemodynamic changes in kidney vessel with an increase in glomerular permeability and a mild degree of inflammation and hypoxia [9,11,15,26]. However, in a meta-analysis study of the effects of exercise training on proteinuria in humans, Yang et al. (2020) [32] found that exercise does not worsen proteinuria in adult CKD patients, although a beneficial effect is uncertain and limited to a narrow time interval comparing proteinuria before and after exercise. In dogs, uAC is a more sensitive marker of reduced renal function than serum creatinine or UPC [29]. In our study, we also found a positive correlation between UPC and urinary albumin and uAC immediately after exercise at T1, but UPC was below the threshold value of 0.2 for healthy dogs, while uAC was found in the range of 0.03 and 0.3, which is considered indicative of microalbuminuria and similar to that reported in military dogs after a training session of search activity [9]. These data confirm that physical exercise induces a transient reduction in renal function resulting in proteinuria, mainly characterized by albuminuria. Furthermore, with the more sensitive silver nitrate staining (Figure 3), we observed at T1, a significantly higher number of bands, suggesting that the proteinuria after exercise is due to urinary of other proteins than albumin. Further studies are needed to identify the LMM proteins in urine after exercise.

SDS-PAGE electrophoresis also allowed us to measure the concentration of urinary uromodulin. Uromodulin is the most abundant urinary protein under physiological conditions in dogs and humans [11,33], produced by renal tubular epithelial cells, released into the lumen by a proteolytic cleavage and excreted in the urine [34]. Low concentrations of uromodulin can also be measured in serum and its decrease in serum or urine is considered a promising marker of CKD [11,35]. In urine, uromodulin tends to form large aggregates and the determination of its concentration by ELISA is subject to serious bias [34]. In contrast, the determination of urinary uromodulin concentration using SDS-PAGE electrophoresis does not present any difficulties and provides additional information on renal function without using invasive samples such as blood. In our study, urinary uromodulin showed values similar to those reported in dogs by Spinella et al. (2023) [9], but after the competition a significantly lower concentration was found with respect to T0, suggesting the presence of a transient tubular impairment affecting the release of uromodulin in the urine. Moreover, uUC was negatively correlated with age at both T0 and at T2, while at T1 was positively correlated with UPC. The decrease in urinary uromodulin has also been previously observed in non-proteinuric stage 1 CKD dogs [11]. The results of our study suggest that the urinary concentration of this protein could be considered as an early biomarker of renal impairment.

## 5. Conclusions

The results obtained show that well-trained healthy dogs should not show any pathological variations in urinary parameters after canicross competition. Despite the non-invasive nature of the sampling procedure and the low cost of the analysis, urinalysis and the evaluation of the urinary proteome by SDS-PAGE electrophoresis are still rarely used as a routine clinical diagnostic tool in young and adult sports dogs, in contrast to human athletes. The authors believe that this preliminary study may provide first scientific evidence that could lead to the inclusion of urine sampling as a routine tool to non-invasively assess the effects of exercise, and consequently the health status of dogs, using urinary biochemical parameters and urinary proteins separated using SDS-PAGE electrophoresis; also supporting the idea that this endurance sport mainly requires dogs trained on resistance capability for a rapid return to physiological values Although our results are statistically supported and clinically promising, we obtained data from a limited number of subjects and from a short post-exercise follow-up. Further studies need to include a larger number of subjects and a follow-up up to 24 h after exercise to monitor a possible biphasic pattern of exercise-induced proteinuria as reported in sedentary and trained men [36].

## Figures and Tables

**Table 1:** The urinary analytes in the urine samples. The data are reported as mean ± SE. In the same row the same superscript number indicates a significant difference between time points (*p* < 0.05).

Analytes	T0	T1	T2
pH	6.6 ± 0.3 ^(1,2)^	8.1 ± 0.2 ^(1)^	8.1 ± 0.2 ^(2)^
USG	1051 ± 6	1041 ± 6	1042 ± 7
Protein mg/dL	40 ± 12	60 ± 12	45 ± 13
Urobilin mg/dL	1.4 ± 1.4	1.4 ± 1.4	0
Bilirubin mg/dL	1.4 ± 1.4	0	1.4 ± 1.4
Glucose mg/dL	21 ± 7	25 ± 10	50 ± 15
Ketones mg/dL	0	0	0
Erythrocytes (+/number of specimens)	+/2	++/1	0
Leucocytes (+/number of specimens)	+/2	0	+/1

USG: urine specific gravity.

**Table 2:** Concentrations of creatinine and total proteins, uromodulin, and albumin, with their ratios in the urine samples. Data are reported as mean ± SE. In the same row the same superscript number indicates a significant difference between time points (*p* < 0.05).

Analytes	T0	T1	T2
uCr mg/dL	255 ± 43	189 ± 22	202 ± 32
uTP mg/dL	20.3 ± 3.3	23.7 ± 2.9	17.3 ± 3.6
UPC	0.080 ± 0.005 ^(1)^	0.128 ± 0.014 ^(1)^	0.081 ± 0.009
Uromudulin mg/dL	9.3 ± 1.5 ^(1)^	6.1 ± 0.7	5.0 ± 0.9 ^(1)^
uUC	0.044 ± 0.011	0.033 ± 0.004	0.030 ± 0.007
Albumin mg/dL	1.5 ± 0.5 ^(1)^	7.7 ± 2.6 ^(1,2)^	2.0 ± 0.7 ^(2)^
uAC	0.006 ± 0.002 ^(1)^	0.063 ± 0.036 ^(1,2)^	0.011 ± 0.003 ^(2)^

uCr: urine creatinine, uTP: urine total proteins; UPC: urine protein:creatinine ratio; uUC: urine uromodulin:creatinine ratio; uAC: urine albumin:creatinine ratio.

**Table 3:** Number of protein bands (reported as mean ± SE) in urines sampled from all specimens at T0, T1 and T2 evidenced in a SDS-PAGE gel 12% stained with silver staining. The same superscript indicates a significant difference between time point.

	Number of LMM Bands
T0	9.1 ± 0.8 ^(1)^
T1	11.6 ± 0.8 ^(1,2)^
T2	9.2 ± 0.5 ^(2)^

LMM: low molecular mass proteins.

## Data Availability

All data reported in this study are available at https://amsacta.unibo.it/id/eprint/7805/ (accessed on 3 November 2024).

## Supplementary Materials

The following supporting information can be downloaded at: https://www.mdpi.com/article/10.3390/ani14223216/s1, Table S1: Prediction equations and parameters of regression models; Table S2: The urinary analytes in the urine samples of dogs grouped as younger adult and older adult dogs and as entire and spayed female dogs; Table S3: Concentrations of total proteins, creatinine, albumin, uromodulin and their ratios in the urine samples of dogs grouped as younger adults and older adults and as entire and spayed dogs; Table S4: Matrix Spearman correlation at each time.

## References

[1] Lafuente P., Whyle C. (2018). A Retrospective Survey of Injuries Occurring in Dogs and Handlers Participating in Canicross. Vet. Comp. Orthop. Traumatol..

[2] Erjavec V., Vovk T., Nemec Svete A. (2022). The Effect of Two Acute Bouts of Exercise on Oxidative Stress, Hematological, and Biochemical Parameters, and Rectal Temperature in Trained Canicross Dogs. Front. Vet. Sci..

[3] Pechette M.A., Shoben A.B., Kieves N.R. (2021). Internet-based survey of the frequency and types of orthopedic conditions and injuries experienced by dogs competing in agility. J. Am. Vet. Med. Assoc..

[4] Tomlinson J.E., Manfredi J.M. (2018). Return to Sport after Injury: A Web-Based Survey of Owners and Handlers of Agility Dogs. Vet. Comp. Orthop. Traumatol..

[5] Cullen K.L., Dickey J.P., Bent L.R., Thomason J.J., Moëns N.M. (2013). Internet-based survey of the nature and perceived causes of injury to dogs participating in agility training and competition events. J. Am. Vet. Med. Assoc..

[6] Koh R., Montalbano C., Gamble L.J., Walden K., Rouse J., Liu C.C., Wakshlag L.G., Wakshlag J.J. (2020). Internet survey of feeding, dietary supplement, and rehabilitative medical management use in flyball dogs. Can. Vet. J..

[7] Pinto K.R., Chicoine A.L., Romano L.S., Otto S.J.G. (2021). An Internet survey of risk factors for injury in North American dogs competing in flyball. Can. Vet. J..

[8] Montalbano C., Gamble L.J., Walden K., Rouse J., Mann S., Sack D., Wakshlag L.G., Shmalberg J.W., Wakshlag J.J. (2019). Internet Survey of Participant Demographics and Risk Factors for Injury in Flyball Dogs. Front. Vet. Sci..

[9] Spinella G., Valentini S., Matarazzo M., Tidu L., Ferlizza E., Isani G., Andreani G. (2023). Effects of exercise on urinary biochemical parameters and proteins in a group of well-trained military dogs. Vet. Q..

[10] Ferlizza E., Campos A., Neagu A., Cuoghi A., Bellei E., Monari E., Dondi F., Almeida A.M., Isani G. (2015). The effect of chronic kidney disease on the urine proteome in the domestic cat (*Felis catus*). Vet. J..

[11] Ferlizza E., Isani G., Dondi F., Andreani G., Vasylyeva K., Bellei E., Almeida A.M., Matzapetakis M. (2020). Urinary proteome and metabolome in dogs (*Canis lupus familiaris*): The effect of chronic kidney disease. J. Proteom..

[12] Daniel W.W., Cross C.L. (2019). Biostatistics a Foundation for Analysis in the Health Sciences.

[13] Fox J., Monette G. (1992). Generalized collinearity diagnostics. JASA.

[14] Bouyer J., Hémon D., Cordier S., Derriennic F., Stucker I., Stengel B., Clavel J. (2009). Epidemiologie Principes et Méthodes Quantitatives.

[15] Bacic A., Kogika M.M., Barbaro K.C., Iuamoto C.S., Simões D.M., Santoro M.L. (2010). Evaluation of albuminuria and its relationship with blood pressure in dogs with chronic kidney disease. Vet. Clin. Pathol..

[16] Lopedote M., Valentini S., Musella V., Vilar J.M., Spinella G. (2020). Changes in Pulse Rate, Respiratory Rate and Rectal Temperature in Working Dogs before and after Three Different Field Trials. Animals.

[17] Rovira S., Muñoz A., Benito M. (2007). Hematologic and biochemical changes during canine agility competitions. Vet. Clin. Pathol..

[18] Constable P.D., Hinchcliff K.W., Olson J.L., Stepien R.L. (2000). Effects of endurance training on standard and signal-averaged electrocardiograms of sled dogs. Am. J. Vet. Res..

[19] McGowan R.T.S., Rehn T., Norling Y., Keeling L.J. (2014). Positive affect and learning: Exploring the “Eureka Effect” in dogs. Anim. Cogn..

[20] Van Paridon K.N., Timmis M.A., Nevison C.M., Bristow M. (2017). The anticipatory stress response to sport competition; a systematic review with meta-analysis of cortisol reactivity. BMJ Open Sport Exerc. Med..

[21] Rishniw M., Bicalho R. (2015). Factors affecting urine specific gravity in apparently healthy cats presenting to first opinion practice for routine evaluation. J. Fel. Med. Sur..

[22] Rudinsky A., Cortright C., Purcell S., Cordner A., Lord L., Wellman M., Di Bartola S., Chew D. (2019). Variability of first morning urine specific gravity in 103 healthy dogs. Vet. Int. Med..

[23] McGlynn A., Mrofchak R., Madan R., Madden C., Jahid M.J., Mollenkopf D., Wittum T., Justice S.S., Rudinsky A., Hokamp J. (2023). Longitudinal examination of urine pH, specific gravity, protein, culture, and antimicrobial resistance profiles in healthy dogs. J. Vet. Intern. Med..

[24] van Vonderen I.K., Kooistra H.S., Rijnberk A. (1997). Intra- and interindividual variation in urine osmolality and urine specific gravity in healthy pet dogs of various ages. J. Vet. Intern. Med..

[25] Chew D.J., Di Bartola S.P., Schenck P.A., Chew D.J. (2011). Canine and Feline Nephrology and Urology.

[26] Pasławska U., Szczepankiewicz B., Bednarska A., Pasławski R. (2020). Impact of High Temperature on Post-Exercise Albuminuria in Dogs. Animals.

[27] Pero R., Brancaccio M., Mennitti C., Gentile L., Arpino S., De Falco R., Leggiero E., Ranieri A., Pagliuca C., Colicchio R. (2020). Urinary biomarkers: Diagnostic tools for monitoring athletes’ health status. Int. J. Environ. Res. Public Health.

[28] Kuwahara Y., Nishii N., Takasu M., Ohba Y., Maeda S., Kitagawa H. (2008). Use of urine albumin/creatinine ratio for estimation of proteinuria in cats and dogs. J. Vet. Med. Sci..

[29] Paukner K., Filipejova Z., Mareš J., Vávra M., Rehakova K., Proks P., Gabriel V., Crha M. (2024). A comprehensive analysis of albuminuria in canine chronic kidney disease. Vet. Med. Sci..

[30] International Renal Interest Society (IRIS) Staging System. http://www.iris-kidney.com/.

[31] Falus F.A., Vizi Z., Szabò K.E., Muller L., Reiczigel J., Balogh N., Manczur F. (2022). Establishment of a reference interval for urinary albumin to creatinine ratio in dogs. Vet. Clin. Pathol..

[32] Yang L., Wu X., Wang Y., Wang C., Hu R., Wu Y. (2020). Effects of exercise training on proteinuria in adult patients with chronic kidney disease: A systematic review and meta-analysis. BMC Nephrol..

[33] Schaeffer C., Devuyst O., Rampoldi L. (2021). Uromodulin: Roles in Healthand Disease. Annu. Rev. Physiol..

[34] Seo D., Yang Y., Hwang S.H., Jung J.H., Cho S., Choi G., Kim Y. (2022). Serum uromodulin in dogs with chronic kidney disease. J. Vet. Intern. Med..

[35] Scherberich J.E., Gruber R., Nockher W.A., Christensen E.I., Schmitt H., Herbst V., Block M., Kaden J., Schlumberger W. (2018). Serum uromodulin-a marker of kidney function and renal parenchymal integrity. Nephrol. Dial. Transplant..

[36] Sentürk U.K., Kuru O., Koçer G., Gündüz F. (2007). Biphasic pattern of exercise-induced proteinuria in sedentary and trained men. Nephron Physiol..

