# Supplementary material for: Effects of Exercise on Urinary Parameters and Proteins in Well-Trained Canicross Dogs: A Preliminary Study
Source: Animals (Basel). 2024 Nov 9;14(22):3216. doi: 10.3390/ani14223216 (PMC11590915; doi:10.3390/ani14223216)
Supplement: Supplementary file 1 [file animals-14-03216-s001.zip › animals-3285860-supplementary.pdf]

Table S1. Prediction equations and parameters of regression models

Regression model	Variables	b estimates coefficient	coefficient p-value	VIF multicollinearity	F statistic p-value	Shapiro-Wilk p-value of residuals	Breusch-Pagan p-value of residuals	R^2 adjusted	R^2
Uromodulin(T2)=b_0+Albumin(t0)+Albumin(T1)+uUC(T1)+Ɛ	Intercept	-1.0339	0.7161	-	0.04578	0.3827	0.320	0.637	-
	Albumin(t0)	1.0386	0.0494	1.033987					
	Albumin(t1)	-0.5121	0.0171	4.074361					
	uUC(t1)	254.1392	0.0444	4.069886					
Albumin(T2)=b_0+uAC(t0)+Ɛ	Intercept	-0.1771	0.596268	-	0.0001	0.9232	0.248	-	0.883
	uAC(t0)	344.244	0.000105	1.594118					
uTP(T2)=b_0+uAC(T0)+USG(T1)+Ɛ	Intercept	-341.5063	0.01117	-	0.01256	0.2429	0.633	0.69	-
	uAC(t0)	846.98479	0.07661	1.005667					
	USG(t1)	0.33945	0.00969	1.005667					
uCr(T2)=b_0+UPC(T0)+Albumin(T0)+Albumin(T1)+Ɛ	Intercept	-180.173	0.1415	-	0.02525	0.1253	0.360	0.716	-
	UPC(t0)	4976.799	0.0151	1.015649					
	Albumin(t0)	15.975	0.1584	1.007765					
	Albumin(t1)	-5.232	0.0313	1.012782					

uCR: urine creatinine; uTP: urine total proteins; UPC: urine protein:creatinine ratio; uUC: urine uromodulin:creatinine ratio; uAC: urine albumin:creatinine ratio; USG: urinary specific gravity; VIF: variance inflation factor

Table S2. The urinary analytes in the urine samples of dogs grouped as younger adult and older adult dogs and as entire and spayed female dogs. The data are reported as mean ± SE.

Analytes	T0	T1	T2	T0	T1	T2	T0	T1	T2	T0	T1	T2
	Younger adult n=7			Older adult n=5			Entire n=7			Spayed n=5		
pH	6.6±0.4	8.0±0.3	8.1±0.4	6.3±0.7	8.2±0.4	8.1±0.3	6.6±0.5	8.1±0.3	7.9±0.4	6.6±0.5	8.1±0.3	8.4±0.5
USG	1052±8	1045±9	1046±9	1050±7	1037±8	1035±12	1045±9	1033±6	1038±11	1060±8	1053±9	1048±5
Protein mg/dL	41±16	46±15	48±17	78±56	80±20	40±21	31±13	51±18	32±14	92±54	72±18	65±20
Urobilin mg/dL	2.4±2.4	2.4±2.4	0	0	0	0	2.4±2.4	0	0	0	3.4±3.4	0
Bilirubin mg/dL	2.4±2.4	0	2.4±2.4	0	0	0	2.4±2.4	0	2.4±2.4	0	0	0
Glucose mg/dL	21±10	29±15	42±20	20±12	20±12	62±24	21±10	43±13	67±16	20±12	0	25±25
Ketones mg/dL	1.4±1.4	0	0	0	0	0	0	0	0	2.0±2.0	0	0
Erythrocytes (+/number of specimens)	+/2	++/1	0	0	0	0	+/2	++/1	0	0	0	0
Leucocytes (+/number of specimens)	+/1	0	0	+/1	0	0	+/2	0	+/1	0	0	0

USG: urine specific gravity

Table S3. Concentrations of total proteins, creatinine, albumin, uromodulin and their ratios in the urine samples of dogs grouped as younger adults and older adults and as entire and spayed dogs. The data are reported as mean ± SE. uCR: urine creatinine, uTP: urine total proteins; UPC: urine protein:creatinine ratio; uUC: urine uromodulin:creatinine ratio; uAC: urine albumin:creatinine ratio

Analytes	T0	T1	T2	T0	T1	T2	T0	T1	T2	T0	T1	T2
	Younger adult n=7			Older adult n=5			Entire n=7			Spayed n=5		
uTP mg/dL	19.8±4.4	23.7±3.6	19.4±4.4	21.1±6.0	23.5±5.9	14.4±5.0	19.6±5.8	23.6±4.9	15.2±4.5	21.4±3.4	23.8±3.6	20.2±4.7
uCr mg/dL	237±58	182±31	222±43	281±75	199±40	177±39	249±76	163±33	197±49	265±36	225±28	208±28
UPC	0.089±0.005	0.141±0.022	0.082±0.004	0.068±0.007	0.108±0.016	0.078±0.018	0.080±0.009	0.142±0.023	0.071±0.005	0.080±0.003	0.106±0.011	0.094±0.015
Uromudulin mg/dL	10.5±2.1	6.1±0.8	4.4±1.6	7.8±2.3	6.1±1.5	5.8±1.2	10.0±2.6	5.7±1.1	4.8±1.3	8.4±0.8	6.5±0.9	5.2±1.4
uUC	0.059±0.017	0.037±0.006	0.020±0.005	0.024±0.007	0.028±0.005	0.044±0.014	0.052±0.019	0.036±0.007	0.034±0.012	0.034±0.005	0.029±0.002	0.024±0.005
Albumin mg/dL	1.1±0.5	9.8±4.3	1.2.0±0.4	2.0±0.9	4.8±1.6	2.0±1.1	1.6±0.7	10.3±4.2	2.2±1.0	1.2±0.7	4.1±1.4	1.7±1.7
uAC	0.006±0.003	0.093±0.061	0.009±0.004	0.006±0.003	0.021±0.005	0.014±0.006	0.007±0.003	0.095±0.061	0.014±0.004	0.005±0.003	0.019±0.007	0.008±0.006

uTP: urine total proteins; uCR: urine creatinine, UPC: urine protein:creatinine ratio; uUC: urine uromodulin:creatinine ratio; uAC: urine albumin:creatinine ratio

Table S4. Matrix Spearman correlation at each time; statistically significant p values are highlighted in bold

		Uromodulin	Albumin	uTP	uCr	uUC	uAC	UPC	Age years
Matrix Spearman correlation p-value T0	Uromodulin		0.3017	0.08	0.0676	0.1803	0.6517	0.8368	0.4715
	Albumin	0.3017		0.091	0.0945	0.9218	**0.0002**	0.8699	0.7984
	uTP	0.08	0.091		**0.0001**	0.3334	0.6023	0.9267	0.6776
	uCr	0.0676	0.0945	**0.0001**		0.3046	0.6918	0.6451	0.4702
	uUC	0.1803	0.9218	0.3334	0.3046		0.612	0.2786	**0.0442**
	uAC	0.6517	**0.0002**	0.6023	0.6918	0.612		0.803	0.9757
	UPC	0.8368	0.8699	0.9267	0.6451	0.2786	0.803		**0.0059**
	Age years	0.4715	0.7984	0.6776	0.4702	**0.0442**	0.9757	**0.0059**	
Matrix Spearman correlation p-value T1		Uromodulin	Albumin	uTP	uCr	uUC	uAC	UPC	Age years
	Uromodulin		0.5363	**0.0203**	**0.0008**	0.0676	0.9314	0.7063	0.8006
	Albumin	0.5363		0.1426	0.7358	0.1064	**0.0001**	**0.0065**	0.553
	uTP	**0.0203**	0.1426		**0.0195**	0.2964	0.3793	0.0795	0.7818
	uCr	**0.0008**	0.7358	**0.0195**		0.6538	0.6515	0.799	0.5092
	uUC	0.0676	0.1064	0.2964	0.6538		0.1924	**0.0334**	0.8032
	uAC	0.9314	**0.0001**	0.3793	0.6515	0.1924		**0.0058**	0.4709
	UPC	0.7063	**0.0065**	0.0795	0.799	**0.0334**	**0.0058**		0.3985
	Age years	0.8006	0.553	0.7818	0.5092	0.8032	0.4709	0.3985	
Matrix Spearman correlation p-value T2		Uromodulin	Albumin	uTP	uCr	uUC	uAC	UPC	Age years
	Uromodulin		0.4489	0.2191	0.1467	**0.0109**	0.9259	0.4133	0.238
	Albumin	0.4489		0.2104	0.3577	0.6414	**0.0026**	0.3065	0.822
	uTP	0.2191	0.2104		**0.0003**	0.7035	0.9218	**0.0009**	0.3967
	uCr	0.1467	0.3577	**0.0003**		0.5928	0.5803	0.0582	0.3349
	uUC	**0.0109**	0.6414	0.7035	0.5928		0.2607	0.8522	**0.0224**
	uAC	0.9259	**0.0026**	0.9218	0.5803	0.2607		1	0.4252
	UPC	0.4133	0.3065	**0.0009**	0.0582	0.8522	1		0.2361
	Age years	0.238	0.822	0.3967	0.3349	**0.0224**	0.4252	0.2361	

uTP: urine total proteins; uCR: urine creatinine, UPC: urine protein:creatinine ratio; uUC: urine uromodulin:creatinine ratio; uAC: urine albumin:creatinine ratio
